# Surgical treatment of bumblefoot in a captive golden eagle (*Aquila chrysaetos*)

**Published:** 2012

**Authors:** Seyedeh Leila Poorbaghi, Moosa Javdani, Saeed Nazifi

**Affiliations:** 1 *Department of Poultry Sciences, Faculty of Veterinary Medicine, University of Shiraz, Shiraz, Iran; *; 2 *Department of Clinical Sciences, Faculty of Veterinary Medicine, University of Razi, Kermanshah, Iran;*; 3 *Department of Clinical Science, Faculty of Veterinary Medicine, Shiraz University, Shiraz, Iran.*

**Keywords:** Bumblefoot, Golden eagle, Footpad, *Corynebacterium *

## Abstract

The golden eagle is one of the world's largest living birds. Footpad dermatitis, also known as plantar pododermatitis or bumblefoot, is a condition characterized by lesions due to contact with unhealthy "perching" conditions, such as plastic perches, sharp-cornered perches on the ventral footpad of birds. A young female golden eagle (*Aquila chrysaetos*) in Fars province of Iran was presented to veterinary clinics of Shiraz University with clinical signs of lameness. The bird was examined clinically and a variety of complementary diagnostic procedures such as blood analysis, X-ray and bacteriological culture were performed. Then a surgical method was pick out for removing of scab, pus and necrotic tissues from abscess on the plantar aspect of bird's feet and healing the skin of area. After surgery, specific bandage, systemic antibiotics and vitamins were used. *Corynebacterium*, a gram negative bacterium, was isolated in the pus from the abscess. After the surgical operation, swelling in the digital pad reduced, the skin of pad healed and the signs of lameness vanished. To prevent developing bumblefoot, good bedding for proper "perching" conditions is necessary. Additionally, vitamin therapy to promote a healthy integument is advised.

## Introduction

The golden eagle (*Aquila chrysaetos*) is one of the world's largest living birds and a vulnerable species that could become endangered if real protection is not afforded by the concerned bodies.^1^ Like all eagles; it belongs to the family *Accipitridae*. This species was first described by Linnaeus in 1758 as *Falco chrysaetos* and is still ubiquitous, being present in Eurasia, North America and parts of Africa. These birds are dark brown, with lighter golden-brown plumage on their heads and necks. Their wingspan averages over 2 m (7 ft) and their length is about 1 m (3 ft).

Bumblefoot is a general term for any inflammatory or degenerative condition of the avian foot, and may range from a very mild redness or abrasion to chronic, deep-seated abscesses. Bumblefoot is found on the plantar aspect of a bird's feet where it can form an abscess. They look like calluses and they feel hard and typically affect both feet. This condition is a progressive, granulomatous pedal disease primarily affecting large species of raptors maintained in captivity.^2^ Bumblefoot is reported in *Anseriformes*, *Ciconiiformes*, *Faiconiformes*, *Galliformes*, *Passeriformes* and *Strigiformes.*^3^^,^^4^ The disease may develop to arthritis, osteomyelitis and septicemia and could cause mortality up to 100% in roseate spoonbill (an aquatic bird of order *Ciconiiformes*).^5^^,^^6^ The causative agents of bumblefoot are *Staphylococcus* *aureus*, *Escherichia coli *or *Proteus spp*. that can involve bone, tendons sheaths and joints, especially tibiotarsal and stifle joints. Infection with *Staphylococcus* *aureus* are not commonly in other location including sternal bursa, yolk sac, heart, vertebrae and eyelid.^7^ All avian species are susceptible to staphylococcal infections but captives birds kept in cages are more prone to bumblefoot. The infection penetrates the foot from the plantar surface as the integrity of the integument impairs. Predisposing factors include excessively dry or abraded feet in water birds; heaviness and inactivity, unhygienic condition, constantly standing on the same diameter perches and the puncture of the metatarsal pad by an overgrown claw of the first or hind digits.^5^ Besides, some forms of bumblefoot are caused by a vitamin A deficiency. This vitamin promotes appetite, digestion, and also increases resistances to infection.^8^ Budgerigars are particularly susceptible to this type of bumblefoot because seeds are typically low in vitamin A. 

We were aware that there was no case report on the bumblefoot in golden eagle. This paper describes the pathological conditions, diagnosis and treatment of bumblefoot in one female golden eagle.

## Case history

**Fig. 1:**
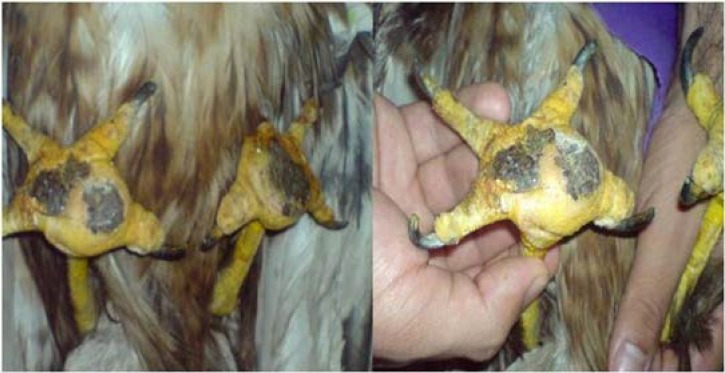
Massive swelling and abscesses in digital pads of both legs in a 2 years old female golden eagle.

A two years old female golden eagle weighing 3 kg with massive swelling abscesses in digital pad of both legs and lameness was presented to the veterinary clinic of Shiraz University in Fars province of Iran (Fig. 1). According to the client's statements, the bird was imprisoned in a small box during past two months. Several complementary diagnostic tests such as blood analysis (differential blood cell count), X-ray and bacteriological culture were performed to identify the cause and extension of infection. Hematological features of this golden eagle are presented in Table 1. 

**Table 1:** Hematological features of the bird with bilateral bumblefoot

**Parameters**	**Case values**	**Normal values**
**Heterophil**	29%	18%
**Lymphocyte**	52%	69%
**Monocyte**	8%	4%
**Eosinophil**	11%	9%

**Fig. 2:**
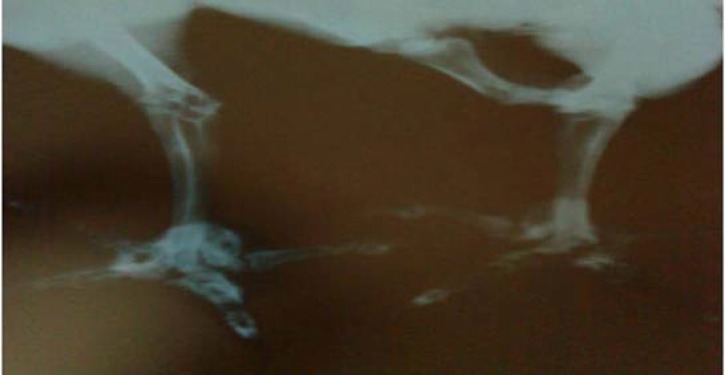
Lateral radiograph shows radiopaque areas in digital pads of both legs, especially in left, that demonstrates pododermatitis

These data demonstrated systemic infection with an increase in heterophils, monocytes and eosinophils accompanied with lymphopenia. Radiographic study of the legs showed radiopaque areas in digital pad (mass of pus) of both legs (especially in left) indicating pododermatitis (Fig. 2). The results of bacteriological culture from drained pus are presented in Table 2. These data demonstrated *Corynebacterium spp.* as of infectious agents in the digital pads potentially leading to bumblefoot.

**Table 2:** Results of pus bacteriological culture from digital pads of one unhealthy golden eagle

**Motility**	**Catalase**	**Oxidase**	**Urease**	**Esculin**	**OF**	**Microscopic appearance**	**Colony on BAP**
-	+	-	Variable	-	No Reaction	Coccorod Gram (+)	Dry & pinpoint colony

OF=Oxidation-Fermentation, BAP=Blood Agar Plate

**Fig. 3:**
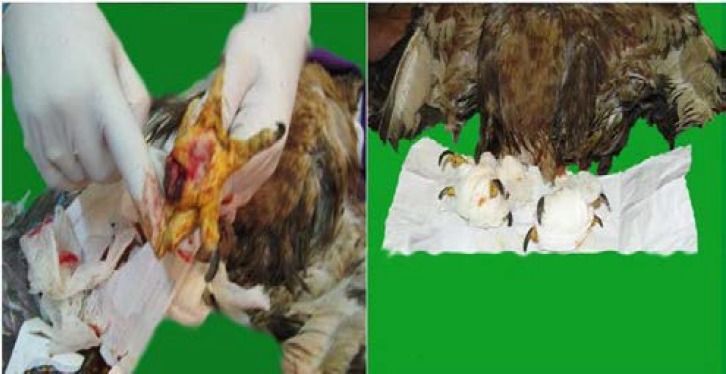
Surgical intervention for wound debridement and drainage

To induce general anesthesia, xylazine 2% (4 mg kg^-1^, Alfasan, Netherlands) and ketamine 10% (5 mg kg^-1^, Alfasan, Netherlands) were used intramuscularly. Wound debridement was carried out to remove scab, pus and necrotic tissues from the abscess on the plantar skin. After surgery, bandage to minimize the pressure on digital pads, systemic antibiotics; penicillin G (100,000 IU kg^-1^, IM, qid, Jaber Ebne Hayyan Co., Iran), ceftizoxime (75 mg kg^-1^, IM, qid, Daanapharma Co., Iran) and vitamins; one dose of AD_3_E (0.3 ml kg^-1^, IM, Erfandarou Co., Iran, containing 80000 IU mL^-1^ vitamin A, 40000 IU mL^-1^ vitamin D and 20 mg mL^-1^ vitamin E) and B-complex (12 mg kg^-1^, IM, Exir Co., Iran), were used for suppress infection and improving general conditions (Fig. 3). After 10 days, skin wound healed and the bird’s general condition improved.

## Discussion

**Fig. 4:**
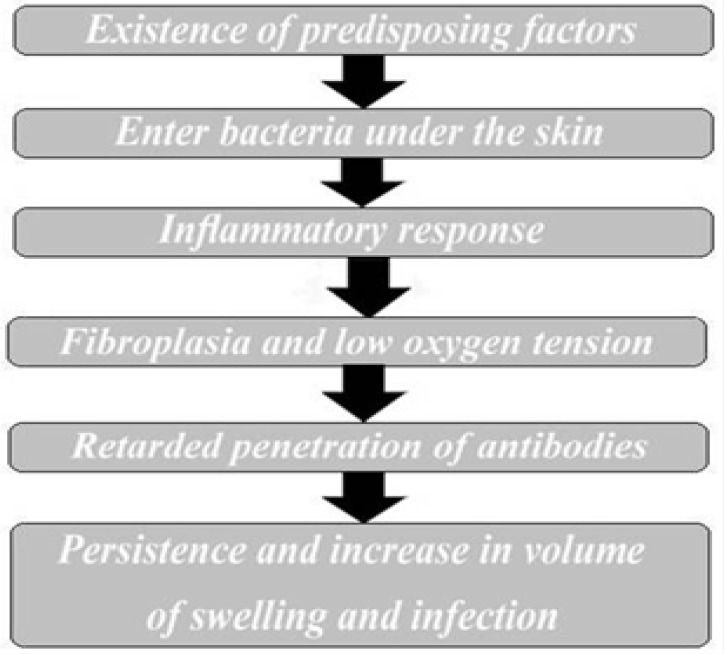
**.** A vicious cycle worsening infection and swelling in bumblefoot

The Golden eagle is one of the endangered largest living birds in the world and if real protection is not afforded, this bird's species will exterminate. Traditionally, the health status of wild populations of birds has been assessed by measurement of population size, reproductive success or annual survival. Published researches on different aspects of golden eagle health are numerous.^1^^,^^9^^-^^15^ Bumblefoot is an infectious and degenerative condition in digital pads and infection can spread into joint and other area in body. Bumblefoot is found on the plantar aspect of a bird's feet where it can form an abscess. There are three different but correlated causes for bumblefoot: Unsuitable perches/ standing and walking platforms, nutritional deficiency, and infections. These abscesses are frequently caused by inappropriate "perching" conditions, such as plastic perches, sharp-cornered perches, the standard perches that tend to come with cages of a uniform diameter, or wire floors. If unsuitable perches or platforms are the cause, a small reddened area, or sometimes a small shiny patch, can usually be seen on the foot.^5^ The condition has been recognized in poultry for many years and also seen in water birds and occasionally in *psittacine* birds mostly in budgerigars. Some forms of bumblefoot are caused by a vitamin A deficiency. Budgerigar is particularly susceptible to vitamin A deficiency because seeds are typically low in vitamin A. This vitamin promotes appetite, digestion, and also increases resistance to infection.^8^ Bacteria, including staphylococcus *spp.* have been identified in some rare cases of bumblefoot, if the wound has not been noticed and treated before it becomes acute. Typically antibiotics, such as erythromycin or penicillin, are prescribed by the vet, if the infection is serious enough. If left untreated, the infection will eventually destroy the bone and travel to other parts of the body. This is a painful condition that can potentially be life endangering. *Staphylococcus aureus*, *Escherichia coli* and *Proteus spp.* detected in caseous or serosanguinous pus extracted from the abscess.^7^ A vicious cycle that worsened the infection and swelling in bumble-foot has been shown in Fig. 4. Treatment and prevention of bumblefoot are dependent to progress of conditions. If the swelling is very small and there is no sign of the infection tracking, only appropriate systemic and local antibiotics (mixed with DMSO to helping the penetration of the drugs) and vitamin therapy will be needed.

In the vast majority of cases, surgery will be necessary. This consists of opening the abscess and carefully removing all caseous and necrotic material, taking care to avoid nerves, tendons and blood vessels.^8^ Remple reported that a four pronged therapeutic regime consisting of (1) systemic antibiotic therapy, (2) direct intralesional antibiotic delivery, (3) surgical debridement, and (4) post-operative protective foot casting has offered the most effective therapy for the majority of bumblefoot cases.^2^ Because of abscess formation with firm capsule around it, the efficacy of systemic antibiotic in treating bumblefoot is ambiguous. However, surgical procedure can empty the abscess, helps to improve the integument and allow antibiotic to penetrate. Therefore, in progressive case of bumblefoot, surgical treatment is needed.

In this case, combination of surgery, local and systemic antibiotic therapy (that choose after antibiogram testing) with good postoperative care were effective and useful to heal the feet abscess.

## References

[1] Nazifi S, Nabinejad A, Sepehrimanesh M (2008). Hematology and serum biochemistry of golden eagle (Aquila chrysaetos) in Iran. Comp Clin Pathol.

[2] Remple JD (2006). A multifaceted approach to the treatment of bumblefoot in raptors. J Exo Pet Med.

[3] Andreasen CB, Saif YM, Barnes HJ, Glisson JR (2003). Staphylococcosis. Disesase of Poultry.

[4] Davidson WR, Netless VF, Couvillion CE (1985). Diseases diagnosed in wild turkeys (Meleagris gallopavo) of the southeastern United States. J Wildlife Dis.

[5] Cooper JE, Needham JR (1976). An investigation into the prevalence of S. aureus on avian feet. Vet Rec.

[6] Marques RMV, de Resende SJ, Donatli VR (2009). A bumblefoot outbreak and fatal septicemia in captive aquatic birds in Brazil. Ciencia Rural Santa Maria.

[7] Andreasen CB, Staphylococcosis ( 2008). Saif YM, Fadly AM, Glisson JR, et al. Disesase of Poultry.

[8] Coles BH (2007). Essential of avian medicine and surgery.

[9] Boeker EL, Ray TD (1971). Golden eagle population studies in the Southwest. Condor.

[10] Brown LH, Watson A (1976). The golden eagle in relation to its food supply. IBIS The international Journal of Avian Science.

[11] Carnie SK (1954). Food habits of nesting golden eagles in the coast ranges of California. The Condor.

[12] Polo FJ, Celdran JF, Peinado VI (1992). Hematological Values for Four Species of Birds of Prey. Condor.

[13] Steenhof K, Kochert MN, McDonald TL (1997). Interactive effects of prey and weather on golden eagle reproduction. J Anim Ecol.

[14] Woodgerd W (1952). Food Habits of the Golden Eagle. J Wildl Manage.

[15] Tjernberg M (1981). Diet of the golden eagle (Aquila chrysaetos) during the breeding season in Sweden. Holarctic Ecol.

